# COVIDSAVIOR: A Novel Sensor-Fusion and Deep Learning Based Framework for Virus Outbreaks

**DOI:** 10.3389/fpubh.2021.797808

**Published:** 2021-11-30

**Authors:** Sharnil Pandya, Anirban Sur, Nitin Solke

**Affiliations:** Symbiosis Institute of Technology, Symbiosis International (Deemed) University, Pune, India

**Keywords:** deep learning, sensor-fusion, COVID-19, novel corona virus, auto-face mask detection, auto-thermal scanning, YOLOv4, body temperature

## Abstract

The presented deep learning and sensor-fusion based assistive technology (Smart Facemask and Thermal scanning kiosk) will protect the individual using auto face-mask detection and auto thermal scanning to detect the current body temperature. Furthermore, the presented system also facilitates a variety of notifications, such as an alarm, if an individual is not wearing a mask and detects thermal temperature beyond the standard body temperature threshold, such as 98.6°F (37°C). Design/methodology/approach—The presented deep Learning and sensor-fusion-based approach can also detect an individual in with or without mask situations and provide appropriate notification to the security personnel by raising the alarm. Moreover, the smart tunnel is also equipped with a thermal sensing unit embedded with a camera, which can detect the real-time body temperature of an individual concerning the prescribed body temperature limits as prescribed by WHO reports. Findings—The investigation results validate the performance evaluation of the presented smart face-mask and thermal scanning mechanism. The presented system can also detect an outsider entering the building with or without mask condition and be aware of the security control room by raising appropriate alarms. Furthermore, the presented smart epidemic tunnel is embedded with an intelligent algorithm that can perform real-time thermal scanning of an individual and store essential information in a cloud platform, such as Google firebase. Thus, the proposed system favors society by saving time and helps in lowering the spread of coronavirus.

## 1. Introduction

The COVID-19 outbreak has given sleepless nights to the entire world for the last 6 months. In recent times, an economy-driven country such as India has recorded more than 1,500,000 cases (1). Considering these facts, researchers are putting enormous efforts into developing innovative solutions to deal with the current pandemic. Furthermore, according to WHO reports, the WHO has put enormous efforts into assisting various developed and developing countries in terms of masks, ventilators, hospital beds, face shields, and other essential health equipments (2). Furthermore, the WHO has also prepared strict guidelines for lock-downs, social-distancing, and testing of COVID-19 suspects. In developing countries, such as India, along with the physical efforts of doctors, nurses, and the paramedical staff, the government has utilized digital technologies in a variety of ways, such as “Aarogya–Setu app,” which can assist citizens in identifying near-by COVID-19 patients using Bluetooth and GPS based remote tracking technologies. However, looking at the impact and the spread of coronavirus, fellow researchers need to put more effort into designing and developing COVID-19 solutions (3–7). It is a fact that fellow researchers have proposed various innovative approaches to deal with the current precarious situation, including automatic sanitizer systems for the disinfection of medical equipment and individuals, thermal scanning guns, and many more. However, the development of COVID-19 assistive systems has remained an open issue for fellow researchers (8–10). In addition to this, various state governments have formulated strict health prevention policies and have made genuine efforts to disinfect various geographical regions using various sanitizers, such as disinfectants and insecticides. The government has also executed numerous health-awareness campaigns for the well-being of society. In some cases, the government has also penalized mischievous individuals who have attempted to break the health and safety procedures. It has also been recorded that the police have to patrol in the surrounding areas to ensure strict adherence to health and safety precautions, such as wearing a mask, maintaining social distancing etc. However, it is also observed and predicted by the world's health experts that society will not have access to coronavirus vaccination in the near future. So the fellow researchers need to keep on developing efficient and effective solutions to avoid unwanted circumstances (11). For this purpose, we have carried out a detailed and rigorous analysis of the existing methodologies and identified certain research gaps. It is also imperative that if the situation continues to persist for a longer time, we have to learn to live with the given problems by keeping ourselves and sound at the workplace. To resolve the discussed issues, in the undertaken study, we have presented a deep learning and sensor-fusion-based approach for detecting a face mask and real-time body temperature for COVID-19, which is term as “Smart Facemask and Thermal scanning kiosk” throughout this paper. The presented deep learning and sensor-fusion-based approach is designed to detect an individual with or without mask conditions and notify security personnel by raising the alarm. Moreover, the smart tunnel is also equipped with a thermal sensing unit embedded with a camera, which can detect the real-time body temperature of an individual concerning the prescribed body temperature limits as prescribed by WHO reports. The presented article is organized as follows: Section 2 discusses the state of the art methodologies. Section 3 discusses the necessity of the presented system, section 4 and 5 discusses the design and experimental setup, architecture design, sensing arrangements, deployments, and the detailed workflow of the presented system. Finally, section 6 provides the concluding remarks.

## 2. Related Work

Yang and his team have proposed an object detection auto-masking neural network for capturing discriminative objects. In this study, a variety of simulation-based study has been carried out. The proposed system is not tested in a real-time environment (12). Joshi R. has presented a health kiosk model using Computational Fluid Dynamics (CFD) simulation-based studies. However, the presented approach has been tested under the simulation environment only (13). Fan and his fellow researchers have proposed an auto-lung segmentation system to detect CT-scans of coronavirus patients. However, the proposed system did not facilitate the scanning of face-masks and the body temperature of humans (14). Maurya and his team have presented an innovative method for disinfecting humans. However, the proposed system did not facilitate any kind of face-mask and body temperature scanning (15). Ghayvat and his team have presented a system to keep the track of social-distancing policies between two persons. For this purpose, the proposed system has used smart cities, such as ITS infrastructures. However, the proposed system did not discuss any kind of scanning approach for the human body and face-masks (2). Abbas and his fellow mates have presented a chest X-ray scanning system of COVID-19 suspects. However, the presented system is not capable to do thermal scanning of a human body or identifying obscured faces (16). Apostolopoulos and Mpesiana have presented a chest X-ray scanning system of COVID-19 suspects. However, the presented systems were not capable to do thermal scanning of a human body or identifying obscured faces (17). Poon and his research team have proposed a system that is capable to carry out obstetric and gynecological scans. The main purpose of the presented system was to do scanning of medical equipment (18). Kim and Lee have presented a respiratory illness scanning system to detect diseases, such as COPD. However, the proposed system did not facilitate thermal scanning and the detection of obscured faces (19). Ucar and fellow researchers have presented an AI-based CT-scan scanning system to detect coronavirus symptoms. However, the presented system did not discuss anything like detecting body temperature or face-masks (20). Kwon and fellow researchers have presented a human scanning system to detect COVID-19 like symptoms for a drive-through. However, the presented system did not discuss anything like detecting body temperature or face-masks (21). Majid et al. (22) researchers have presented a sensor-fusion based wearable device that can scan and disinfect human hands from coronavirus infections (22). Takagi and Yagishita have presented a detailed comparison and discussion of various health and safety policies related to COVID-19 issues. However, the system did not propose any system for COVID-19 like diseases (23). Farman and team have presented a geofencing based real-time tracking system for COVID-19 patients. However, the presented system did not discuss any scanning mechanisms for the current pandemic situations. However, the system was not designed for the disinfection of healthy patients (24). Lippi and fellow researchers have analyzed and discussed numerous bio-safety policies and precautions. However, the presented system did not discuss any scanning approaches for humans (25). Mahammedi et al. (26) have presented a novel solar power-based system to disinfect things such as mobile, key, wallet, and many more. However, the system did not possess the capability to perform any kind of scanning. Pandya et al. (27) have presented a novel face-detection system that can detect a human with fully or partially covered faces. However, the system did not possess the capability to detect face-masks. Ghayvat et al. (28) have presented a sensor-fusion based smart aging system to monitor the elderly well beings. However, the system did not facilitate the scanning of faces and body temperature of a human.

## 3. Necessity of a Smart Face Mask and Thermal Scanning Kiosk

Recent fellow researchers have made efforts to implement a cost-effective pandemic tunnel to disinfect humans from COVID-19 like infections. In this study, an IoT based sensor fusion assistive tunnel has been presented, which can disinfect an individual from the possibility of coronavirus infections. The major contributions of the presented system are as follows: a deep learning and IoT based sensor fusion assistive framework has been proposed to do real-time detection of individuals with face-mask and without face-mask conditions. In the presented research work, a solar-powered smart tunnel has been presented, installed and deployed at the entrance of the Symbiosis institute of technology, Pune. The contributions of the proposed work are as follows:

1. The presented system can also detect an outsider who is entering the building with or without mask conditions using You Only Look Once 4 (YOLOv4) computer vision algorithm (29) and notifies the security control room by raising appropriate alarms.
2. The presented smart face-mask detection and thermal scanning kiosk can function using solar energy during the day, and it functions using a solar power bank at night time. This functionality has been provided by light-dependent resistor (LDR) sensing unit placed in a tunnel.
3. The smart tunnel also provides the facility to do real-time attendance of all the staff entering the smart tunnel, along with thermal scanning and face-mask detection.
4. In the end, web and mobile interface has been designed to provide daily, weekly and monthly reports of the recorded body temperatures of individuals, along with in-out timestamp values. The investigation results validate the performance evaluation of the presented smart face-mask and thermal scanning kiosk. The presented smart tunnel is embedded with an intelligent algorithm that can perform real-time thermal scanning of an individual and stores the essential information in a cloud platform, such as google firebase.

## 4. Design and Experimental Setup

**Figure 1:**
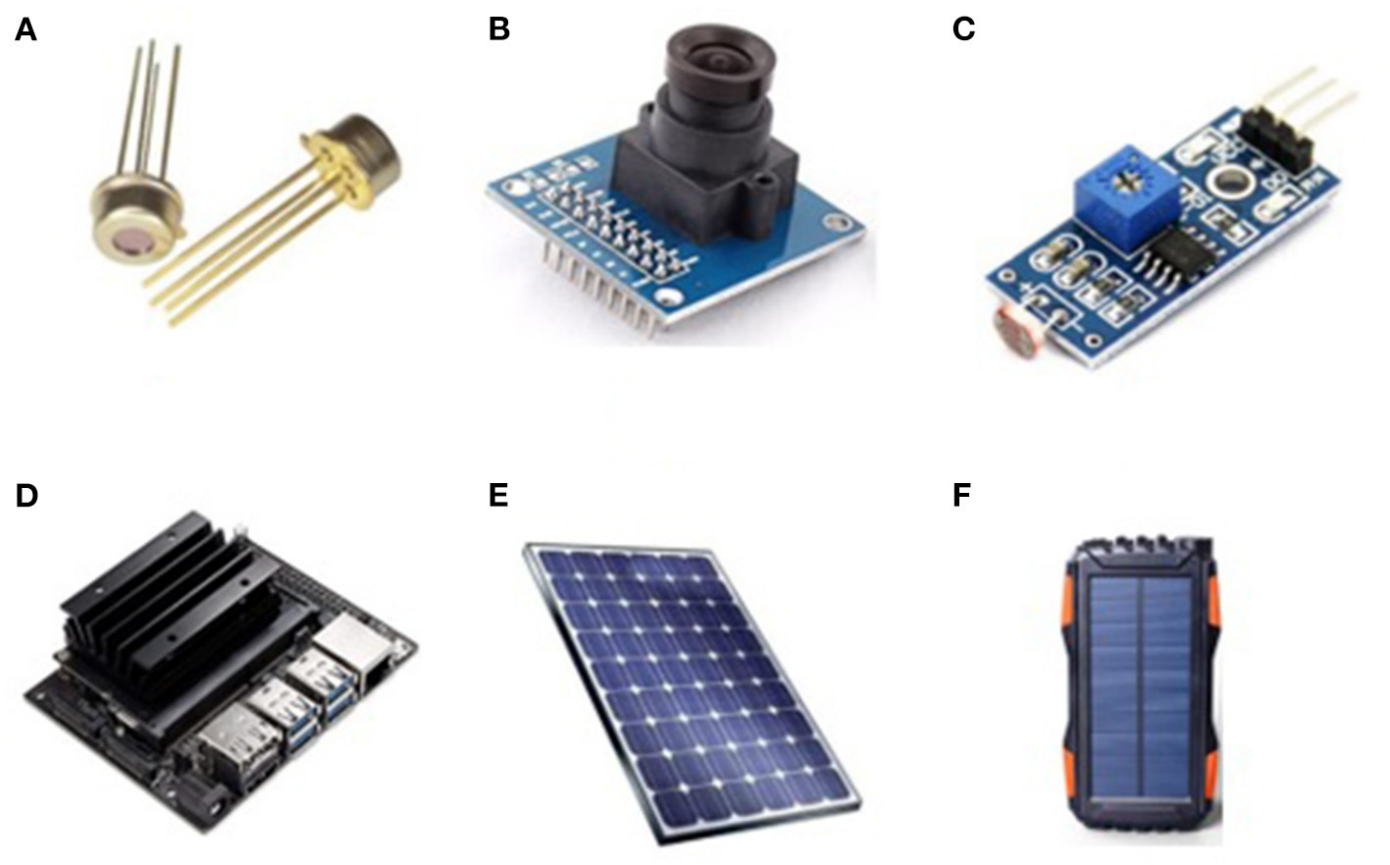
Design and experimental setup of a smart epidemic tunnel **(A)** thermopile temperature sensor a Robocraze OV7670 **(B)** 300KP VGA Camera **(C)** an LDR sensing unit **(D)** a NVIDIA Jetson Nano **(E)** a solar cell **(F)** a solar power bank.

**Figure 2:**
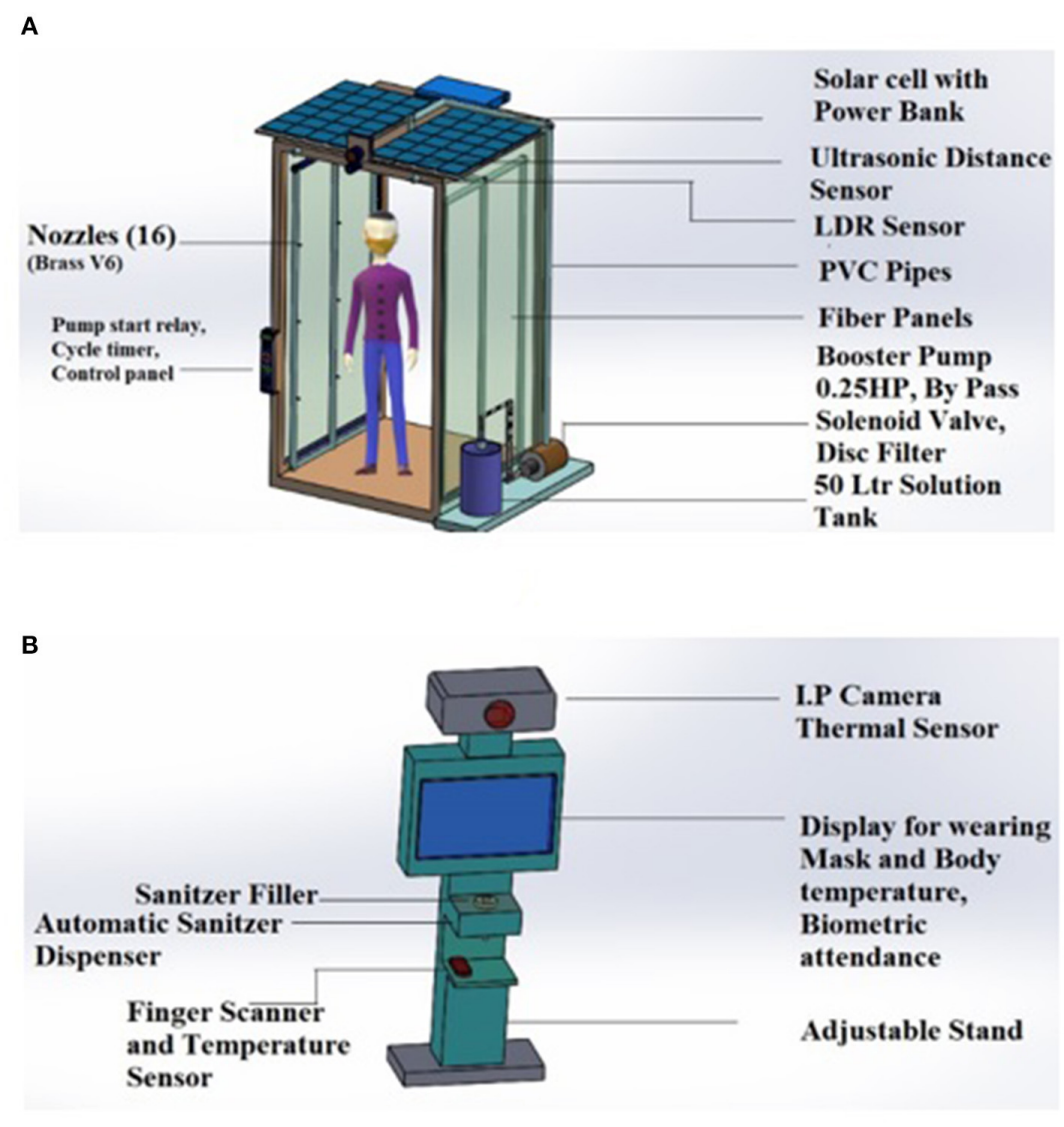
Prototype design of a **(A)** smart epidemic tunnel **(B)** face-mask and thermal scanning Kiosk.

In the experimental setup, each sensor is placed in different positions in the house, with a single ESP8266 attached to it, where the basic data processing takes place. ESP8266 comes with a Wi-Fi module attached to it, using which the processed data is then transferred logged into the Raspberry Pi B+ server. To avoid data duplication of erroneous data, pre-processing of sensor data is done at the ESP8266 level, thus ensuring that Raspberry pi is only used as the local server where clean data is stored in the whole setup. Logged Data is periodically sent to the Cloud storage to ensure backup of data is kept in case of any system failure, along with the logs of whole systems working, which can also be later on utilized for systems debugging. The presented smart tunnel is an extended version of the smart epidemic tunnel, which had facilitated the ultrasonic sensor-based sanitizer system and timestamp-based notifications. In the presented research work, we have proposed a customized smart face-mask and thermal scanning kiosk equipped in a previously presented smart epidemic tunnel. The presented system is flexible enough to be placed outside public spots such as malls, university buildings, companies, bus-stop, hospital ICU units, vegetable markets, railway stations, and many more. Figures 1A–F represent a design and experimental setup used in the conducted experiments such as a Thermopylae sensor, a VGA camera, LDR sensing unit, Netson NVIDIA controller, a solar cell, and a solar power bank. Figure 2 represents a prototype design of the presented smart face mask and thermal scanning kiosk equipped in a smart epidemic tunnel.

## 5. Methodology

Fellow researchers have made efforts to implement a cost-effective pandemic tunnel to disinfect humans from COVID-19 like infections. In this study, an IoT based sensor fusion assistive tunnel has been presented, which can disinfect an individual from the possibility of coronavirus infections. The major contributions of the presented PWP system are as follows: (i) a deep learning and IoT based sensor fusion assistive framework have been proposed to do real-time detection of individuals with face-mask and without face-mask conditions. In the presented research work, a solar-powered smart tunnel has been presented, installed, and deployed at the entrance of the Symbiosis institute of technology, Pune. (i) The presented system can also detect an outsider who is entering the building with or without mask conditions using YOLOv4 computer vision algorithm and notifies the security control room by raising appropriate alarms. (ii) The presented smart face-mask detection and thermal scanning kiosk can function using solar energy during the day, and it functions using a solar power bank at night. This functionality has been provided by an (LDR) sensing unit placed in a tunnel. (iii) The smart tunnel also provides the facility to do real-time attendance of all the staff entering the smart tunnel, along with thermal scanning and face-mask detection. (iv) In the end, web and mobile interface has been designed to provide daily, weekly, and monthly reports of the recorded body temperatures of individuals, along with in-out timestamp values. The investigation results validate the performance evaluation of the presented smart face-mask and thermal scanning kiosk. The presented smart tunnel is embedded with an intelligent algorithm that can perform real-time thermal scanning of an individual and stored the essential information in a cloud platform, such as google firebase. In the pandemic situation, a smart face-mask and thermal scanning kiosk have been presented, equipped in a smart epidemic tunnel. The presented research work is an extended version of the smart epidemic tunnel. In the presented research work, we have added certain novel features to enhance the presented system's performance. The presented deep learning and sensor-fusion based assistive technology (Smart Facemask and Thermal scanning kiosk) will protect the individual using auto face-mask detection and auto thermal scanning to detect the current body temperature. The presented smart tunnel is embedded with an intelligent algorithm that can perform real-time thermal scanning of an individual and stored the essential information in a cloud platform, such as google firebase. Furthermore, in the conducted experiments, the YOLOv4 computer vision algorithm has been applied to detect individuals with face-mask and without faced-mask conditions. Furthermore, the presented system also facilitates a variety of notifications such as an alarm if an individual is not wearing a mask and detects thermal temperature beyond the standard body temperature threshold such as 98.6°F (37°C). Furthermore, a solar power bank is also used for storing solar energy, which the proposed system will utilize at night. In the end, the investigation results validate the performance evaluation of the presented smart face-mask and thermal scanning kiosk.

### 5.1. The Layered Design of a Smart Face-Mask and Thermal Scanning Kiosk

**Figure 3:**
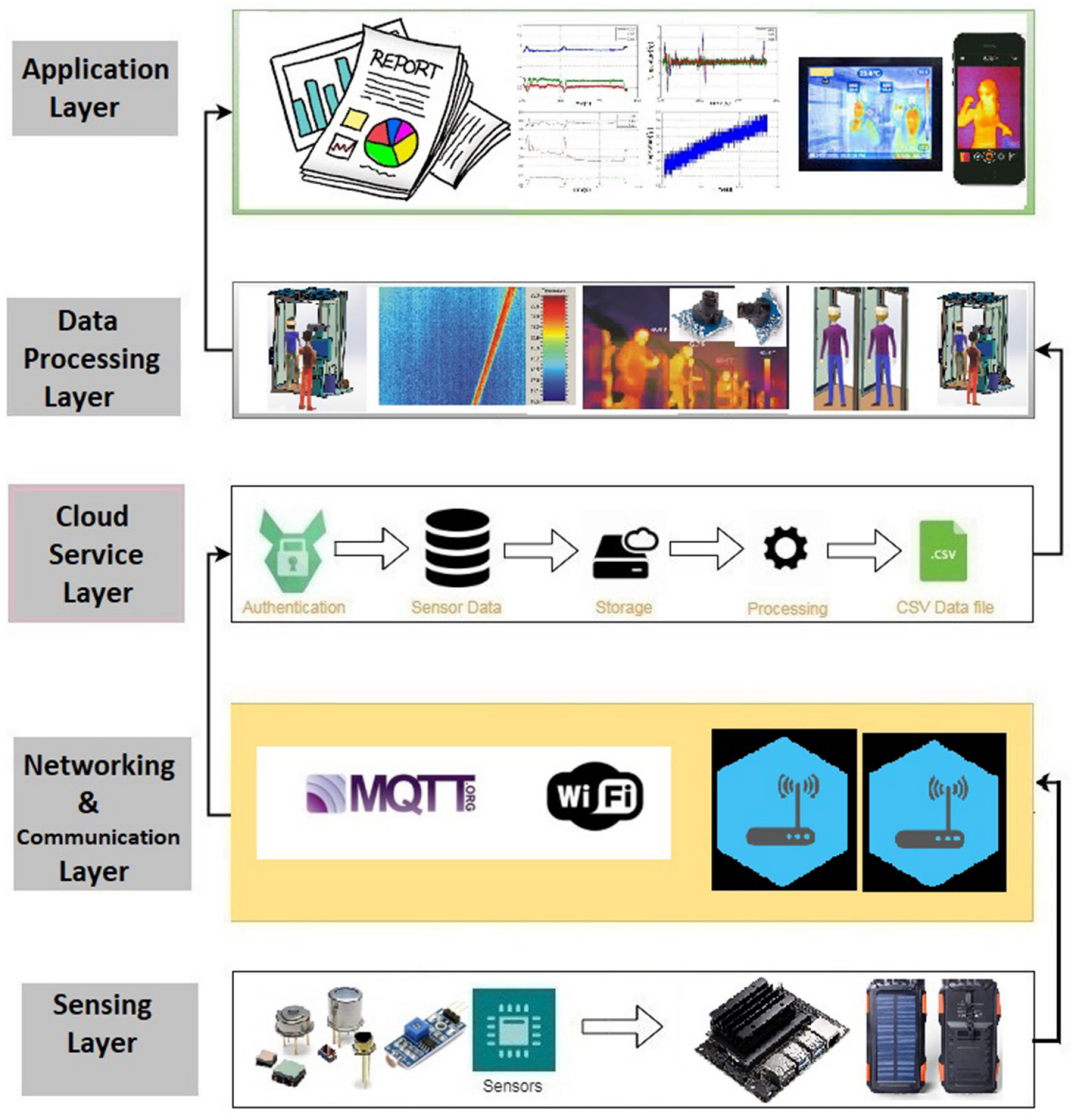
An architectural design of a smart facemask and thermal scanning system prototype design of a facemask and thermal scanning Kiosk.

Figure 3 represents an architectural design of the presented novel deep Learning and sensor-fusion-based approach for detecting a face mask and real-time body temperature for COVID-19 outbreak situations. The extended version of the presented has been placed at the Symbiosis Institute of Technology, Pune, which has been implemented for performing auto-face mask detection and thermal scanning of various Symbiosis stakeholders such as faculty members, students, and external visitors. The presented architectural design contains five layers: (i) physical (sensing) layer, (ii) communication and networking layer, (iii) cloud computing (google firebase storage) layer, (iv) data processing layer, and (v) application layer. The main purpose of the physical sensing layer is to detect the body temperature of individuals who are present in the projection areas of a VGA camera equipped in the presented thermal scanning kiosk. This sensing layer contains various sensing units such as the LDR sensing unit and a thermopile temperature sensor. Furthermore, this layer also contains an intelligent NVIDIA Netson controller, along with a solar cell and a solar power bank. A YOLOv4 computer vision algorithm has been installed in an NVIDIA Netson controller to detect humans with face masks and without face-mask conditions. In addition to this, this layer also facilitates a solar power backup using a solar cell during the day-time and a solar power bank during the night time. Again, in the day-time, the presented smart face-mask and thermal scanning kiosk start functioning using a solar cell.

#### 5.1.1. Sensing Layer

The main purpose of the physical sensing layer is to detect the body temperature of individuals present in the projection areas of a VGA camera equipped in the presented thermal scanning kiosk. This sensing layer contains various sensing units, such as the LDR sensing unit and a thermopile temperature sensor. Furthermore, this layer also contains an intelligent NVIDIA Netson controller, along with a solar cell and a solar power bank. A YOLOv4 computer vision algorithm has been installed in an NVIDIA Netson controller to detect humans with face masks and without face-mask conditions. In addition to this, this layer also facilitates a solar power backup using a solar cell during daytime and a solar power bank during nighttime. Again, in the daytime, the presented smart face-mask and thermal scanning kiosk start functioning using a solar cell.

#### 5.1.2. Communication and Networking Layer

**Figure 4:**
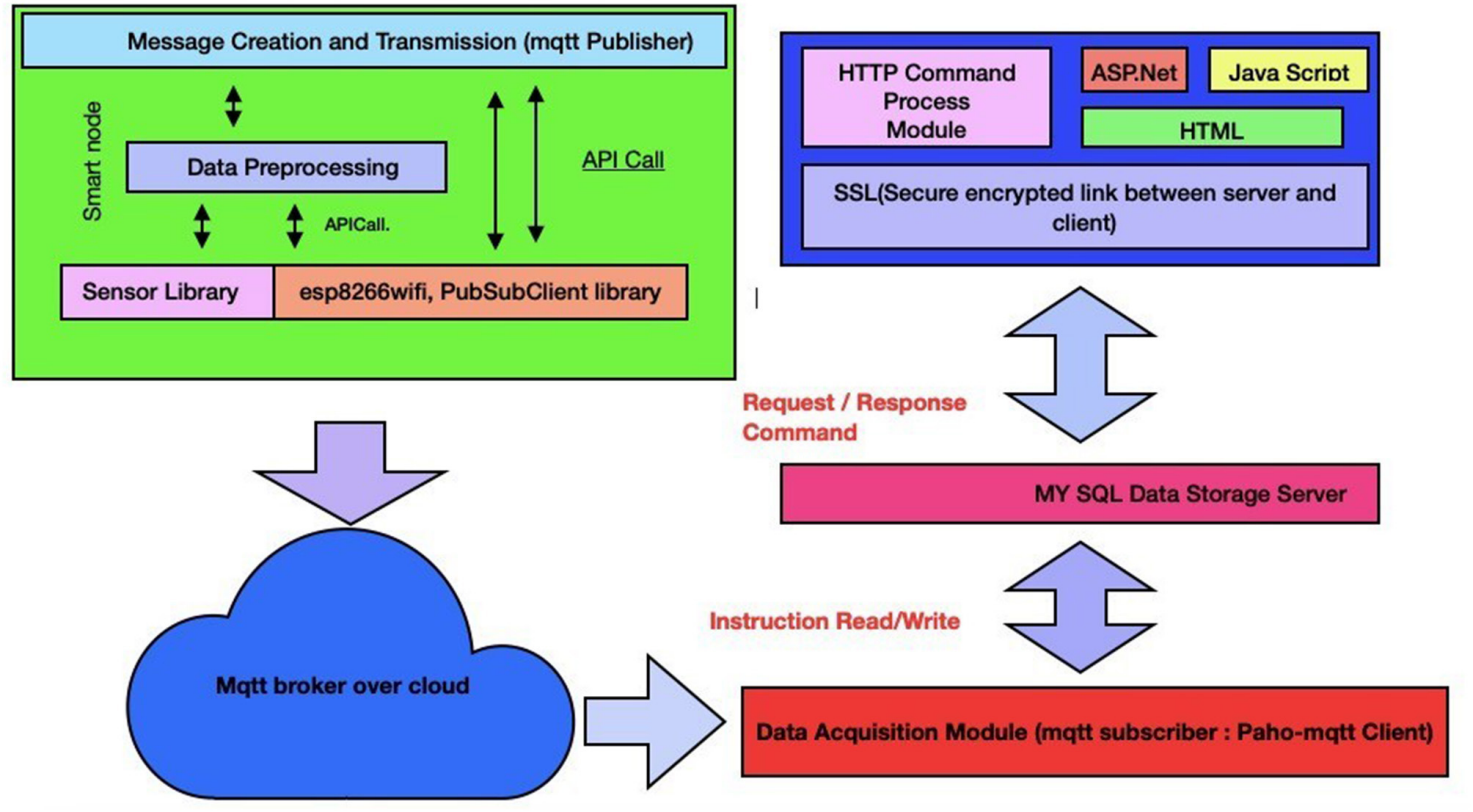
A back-end design of a smart face-mask thermal scanning system.

The networking and communication layer is responsible for interfacing and transmitting information between a physical(sensing) layer, cloud services layer, and an application layer using a broker architecture of an MQTT protocol. Furthermore, it transmits scanned body temperature values, timestamps, and the count of people wearing or not wearing a face mask on a google firebase platform. Figure 4 represents a back-end design of a smart epidemic tunnel.

#### 5.1.3. Communication and Networking Layer

The networking and communication layer is responsible for interfacing and transmitting information between a physical(sensing) layer, cloud services layer, and an application layer using a broker architecture of an MQTT protocol. Furthermore, it transmits scanned body temperature values, timestamps, and the count of people wearing or not wearing a face mask on a google firebase platform. Figure 4 represents a back-end design of a smart epidemic tunnel.

#### 5.1.4. Cloud Computing (Google Firebase) Layer

The cloud computing layer facilitates the storage of the temperature values of individuals scanned using a thermal sensing unit with timestamp values and the count of people with face-mask and without face-mask variations. Furthermore, it keeps track of various individuals who have accessed the smart face-mask and thermal scanning kiosk during the day or night timings. In the conducted experiments, the Google Firebase cloud computing platform has been used for storage purposes.

#### 5.1.5. Processing Layer

The processing layer fetches the temperature and face-mask related values from the Google Firebase database *via* MQTT broker architecture. This layer performs two essential operations: (i) the processing layer detects the body temperature of individuals using a thermal sensor placed in a smart thermal scanning kiosk (ii) it also facilitates two essential bifurcation of the people who are wearing or not wearing face-masks using a YOLOv4 computer vision algorithm deployed in an NVIDIA Netson controller. Eventually, the processed information will be passed on to the application layer for generating various reports.

#### 5.1.6. Application Layer

The application layer received organized information from the processing layer and represented it in various graphical and tabular representations. Furthermore, the application layer also consists of the web and mobile interface, which provides daily, weekly, and monthly updates such as timestamp-based temperature detection reports, the count of people wearing or not wearing face-masks, and the number of individuals who have access to the presented face-mask and thermal scanning kiosk during the day and night timings.

## 6. The Detail Working of the Face-Mask and Thermal Scanning Kiosk

**Figure 5:**
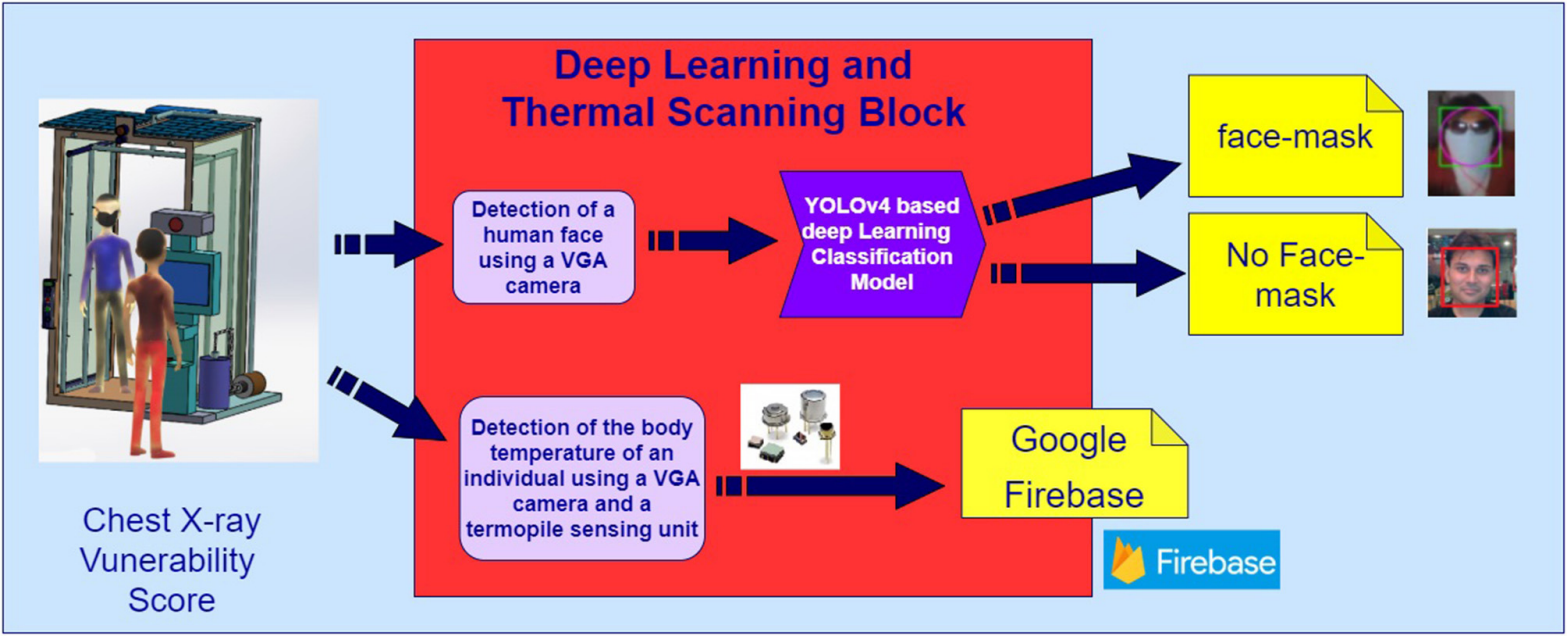
A topology design of a smart facemask and thermal scanning system.

As shown in Figure 5, the presented smart system performs two key operations during this phase: (i) it also facilitates the bifurcation of the people based on a variety of face mask conditions such as with and without face mask conditions using a YOLOv4 computer vision algorithm deployed in an NVIDIA Netson controller. (ii) The presented smart system can detect the body temperature of individuals present in the projection areas of a Robocraze OV7670 300KP VGA camera using a thermopile temperature sensing unit.

### 6.1. The Detailed Working of the Face-Mask Detection Methodology

**Figure 6:**
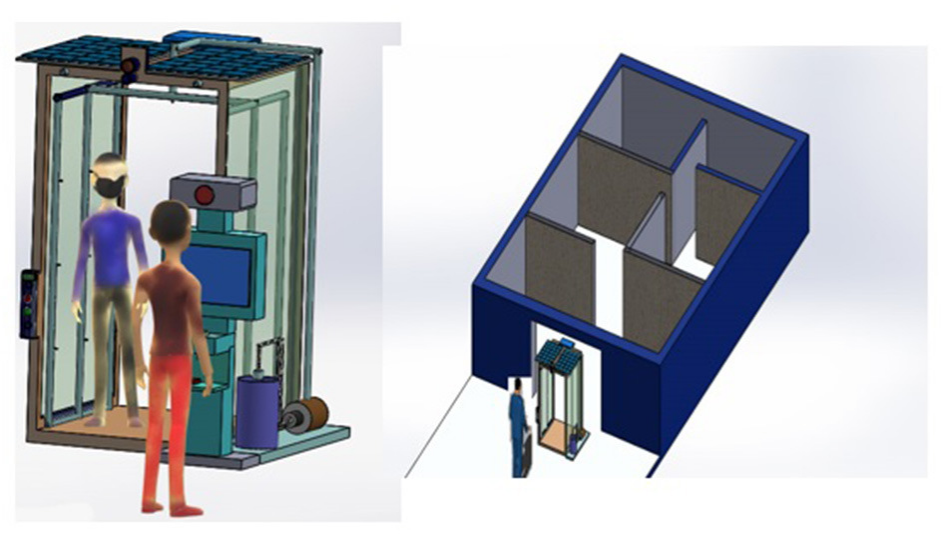
A prototype design of a face-mask detection system using a VGA camera and YOLOv4 deep learning methodology.

**Figure 7:**
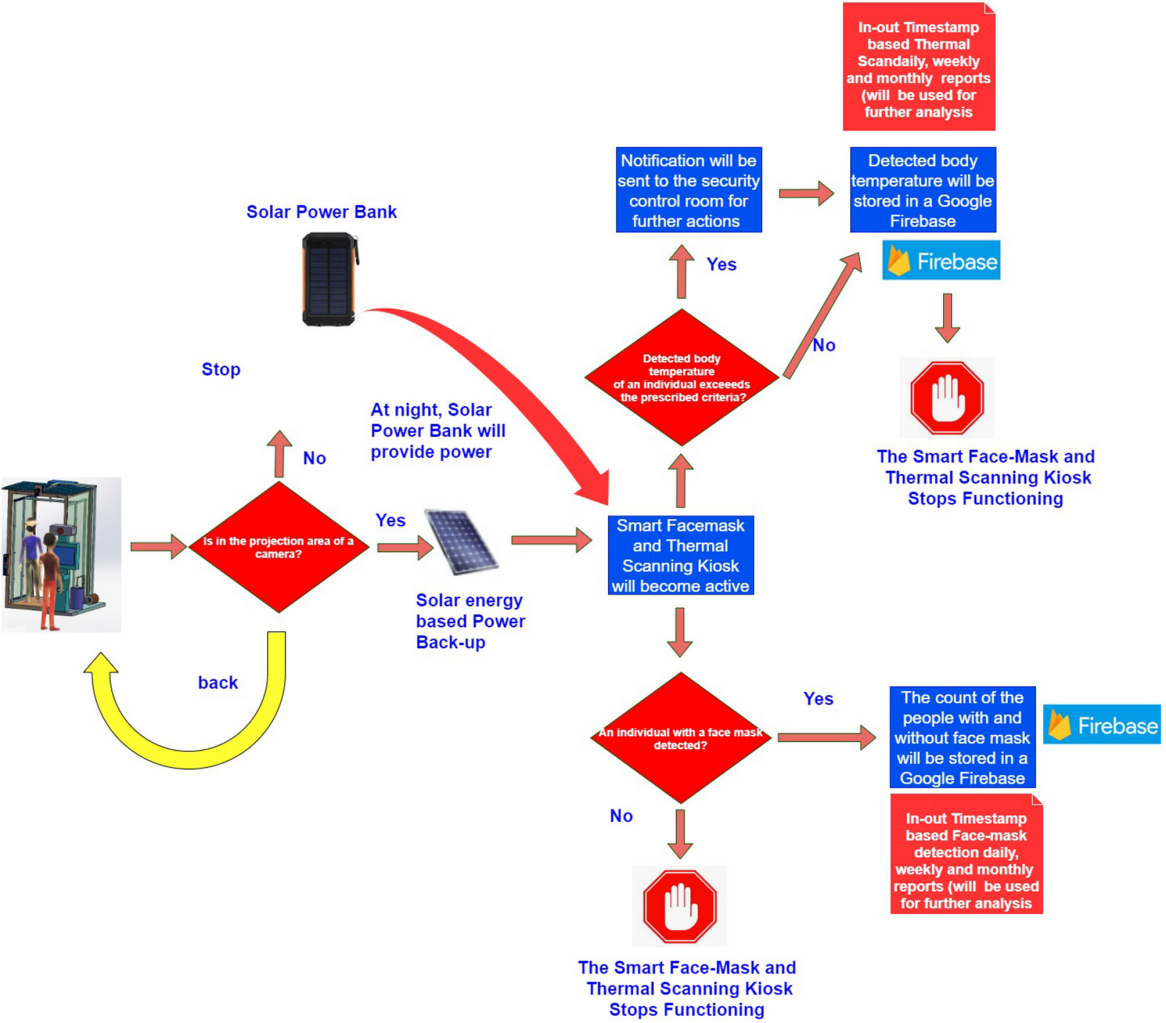
A detailed workflow of a smart face-mask and thermal scanning kiosk.

**Figure 8:**
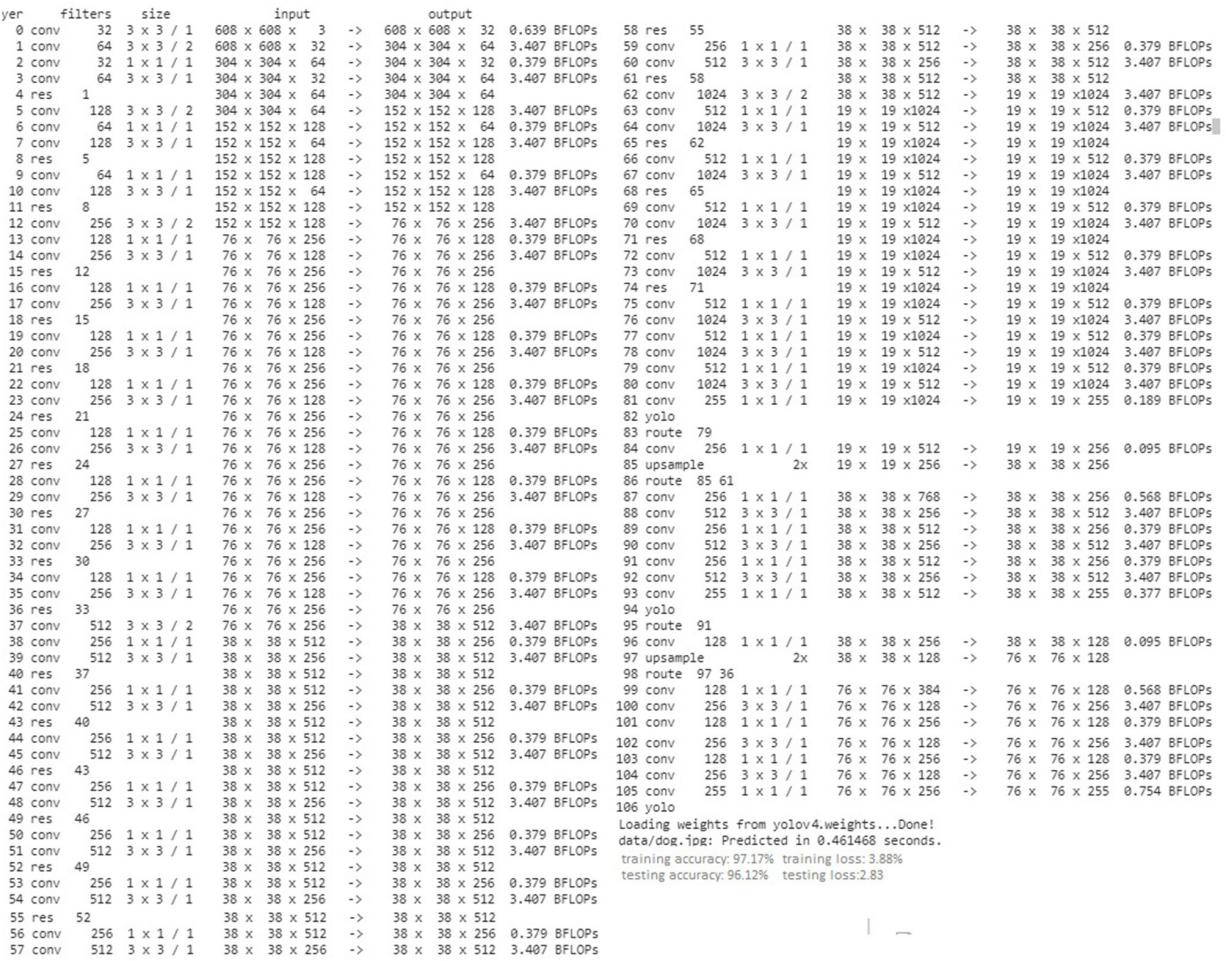
An implementation snapshot of a YOLOv4 deep learning algorithm.

The face-mask detection feature becomes active when an individual comes into the projection area of a VGA camera, as shown in Figure 6. In the conducted experiments, we have used a customized dataset of 1,000 face samples. After an individual is detected by a VGA camera placed in a smart kiosk, a YOLOv4 computer vision algorithm deployed in an NVIDIA Netson controller process the captured frame; during this phase, a YOLOv4 algorithm makes the use of cross-stage partial connection and separates the feature maps in two different parts: (i) the first part of the feature map bypasses the dense block and directly moves toward the transition block. (ii) The second part went through the dense block and reached the transition block. This kind of design increases the efficiency of the YOLOv4 algorithm by reducing the complexity of the presented approach. In addition to this, the YOLOV4 algorithm also utilizes cross-stage-partial connections with CSPDarknet-53 feature extraction methodology, which enables the presented algorithm to achieve very high objection detection accuracy. Figure 7 represents a YOLOv4 methodology, and Figure 8 represents a detailed workflow of the presented smart face-detection and thermal scanning kiosk. As shown in Figure 8, after an individual is detected by a VGA camera placed in a smart kiosk using a YOLOv4 computer vision algorithm, it will process the captured frame of an individual to identify whether the captured individual is wearing a face-mask to protect against COVID-19 or not. Then, the algorithm will transmit the processed information to the Google Firebase and generate various timestamp-based daily, weekly, and monthly reports in both scenarios.

### 6.2. The Detailed Working of the Thermal-Scanning Methodology

The thermal scanning feature of a presented smart kiosk starts functioning when an individual is detected in the projection area of a VGA camera. Once an individual is detected, the presented system will scan their body temperature using a thermal sensor and compare it with the standard body temperature threshold such as 98.6°F (37°C). If the presented kiosk records body temperature of an individual beyond the prescribed limits, it will immediately notify the security control room for further action. Otherwise, timestamp-based records of scanned body temperatures will be stored in Google Firebase for further analysis in both scenarios. The YOLOV4 methodology is divided into two parts: (i) the first part bypasses the dense block and directly moves toward the transition block. (ii) The second part goes through the dense block and reaches the transition block. This kind of design increases the efficiency of the YOLOv4 algorithm by reducing the complexity of the presented approach. In addition to this, the YOLOV4 algorithm also utilizes cross-stage-partial connections with CSPDarknet-53 feature extraction methodology, which enables the presented algorithm to achieve very high objection detection accuracy. Figure 7 represents a YOLOv4 methodology, and Figure 8 represents a detailed workflow of the presented smart face-detection and thermal scanning kiosk.

## 7. Results and Discussions

In this section, a detailed discussion of the obtained results of the presented smart face-mask and thermal scanning kiosk for two different approaches (i) the YOLOv4 based face-mask detection methodology (ii) thermopile temperature sensor VGA camera-based body temperature detection methodology.

### 7.1. Results Discussion of the YOLOv4 Based Face-Mask Detection Methodology

**Figure 9:**
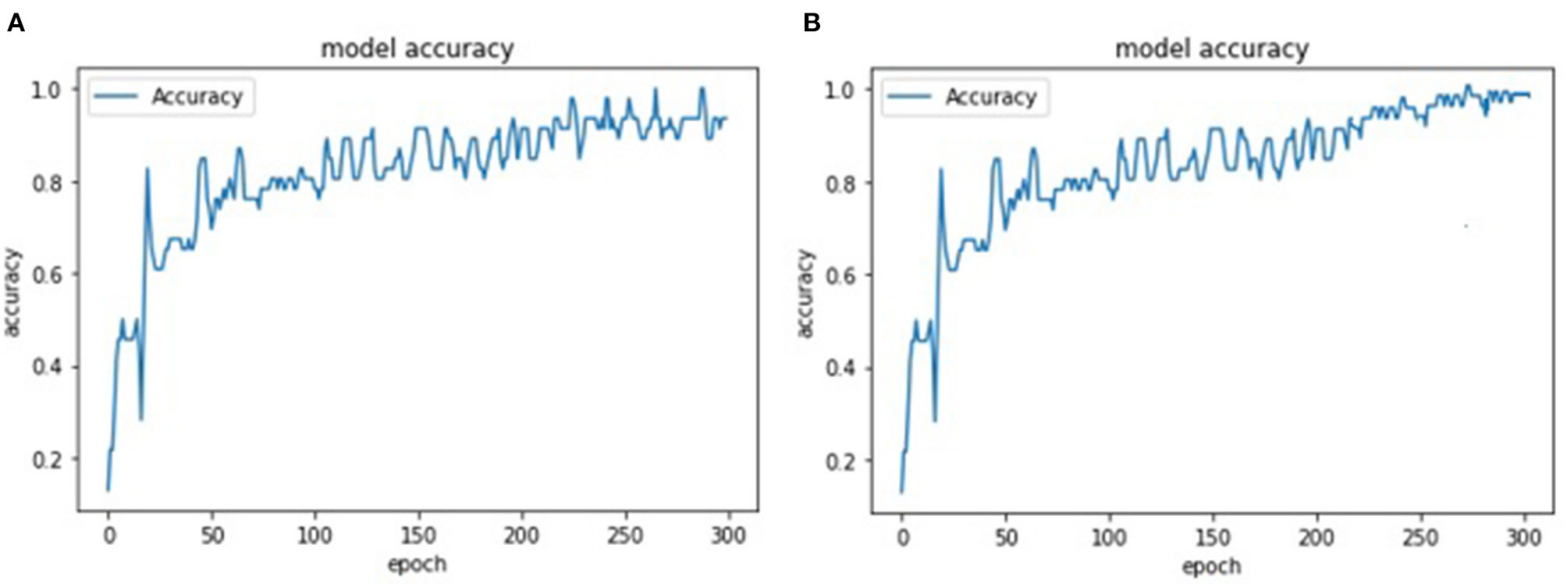
A comparison of a YOLOv4 training and testing accuracy metrics **(A)** training accuracy **(B)** testing accuracy.

**Figure 10:**
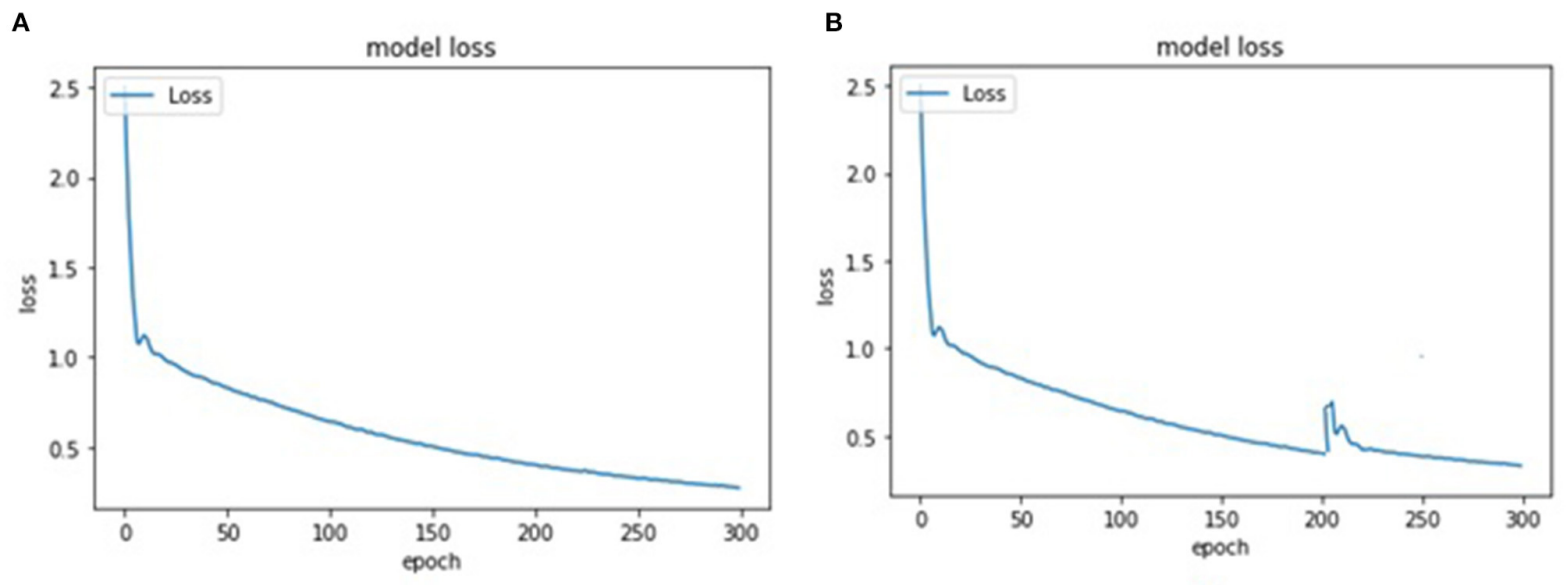
A comparison of a YOLOv4 training and testing accuracy metrics **(A)** training loss **(B)** testing loss.

**Figure 11:**
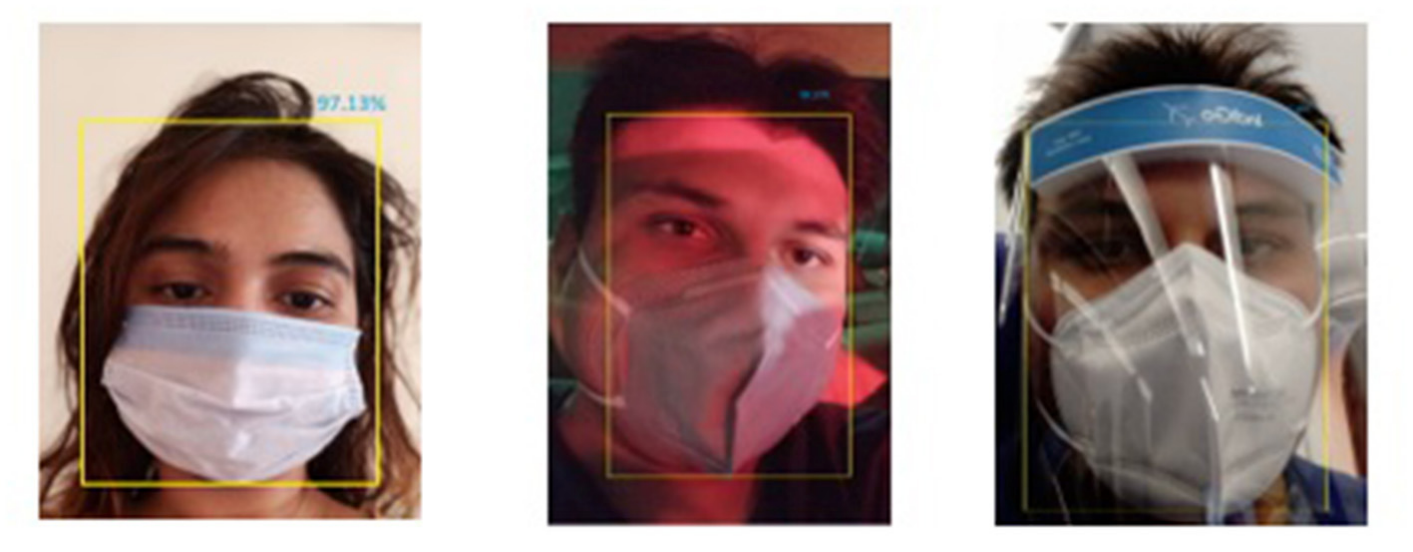
A testing sample of YOLOv4 face-mask detection.

**Figure 12:**
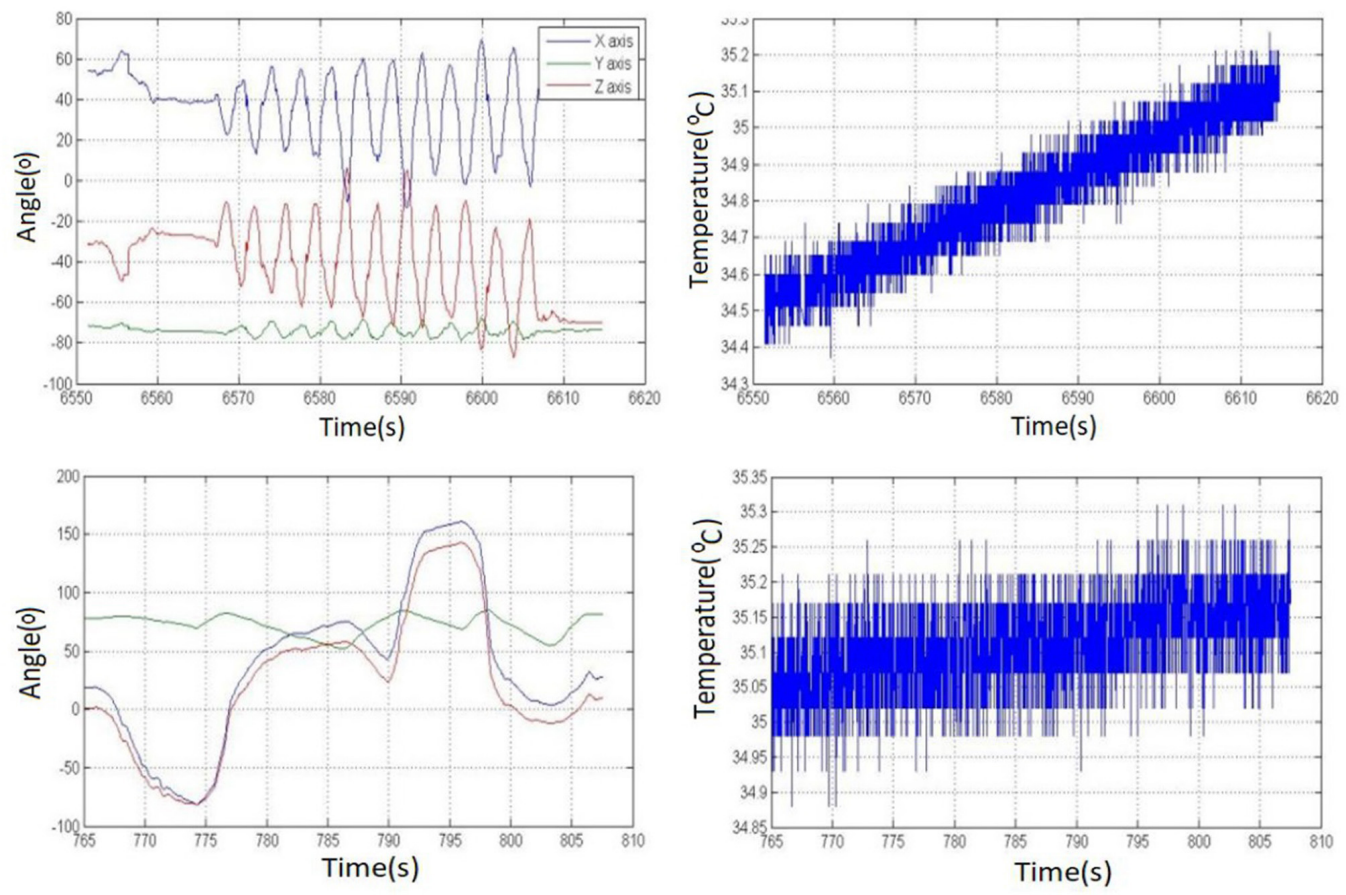
Real-time thermal scanning results of more than 800 individuals.

Figure 9 represents training and testing snapshots of the presented YOLOv4 deep learning methodology. A customized dataset of 1,000 face images was used in the conducted experiments and trained using the YOLOv4 methodology. As described in section 5.2.1, in the conducted experiments, we have used CSPDarknet-53 feature extraction methodology along with the presented YOLOv4 deep learning approach. As shown in Figure 9, we have modified the presented YOLOv4 algorithm to achieve high accuracy in detecting individuals with and without face-mask conditions. Based on the conducted experiments, the presented YOLOV4 methodology has recorded face-mask detection training and testing accuracy of 97.17 and 96.12%, as shown in Figure 10. As shown in Figure 11, based on the conducted experiments, the presented YOLOv4 deep learning algorithm has recorded very minimal training and testing loss, around 1.83 and 2.88%, respectively. Figure 12 depicts face-mask detection samples detected using the YOLOv4 algorithm.

### 7.2. Results Discussion of the Thermal Scanning Based Body Temperature Detection Methodology

**Figure 13:**
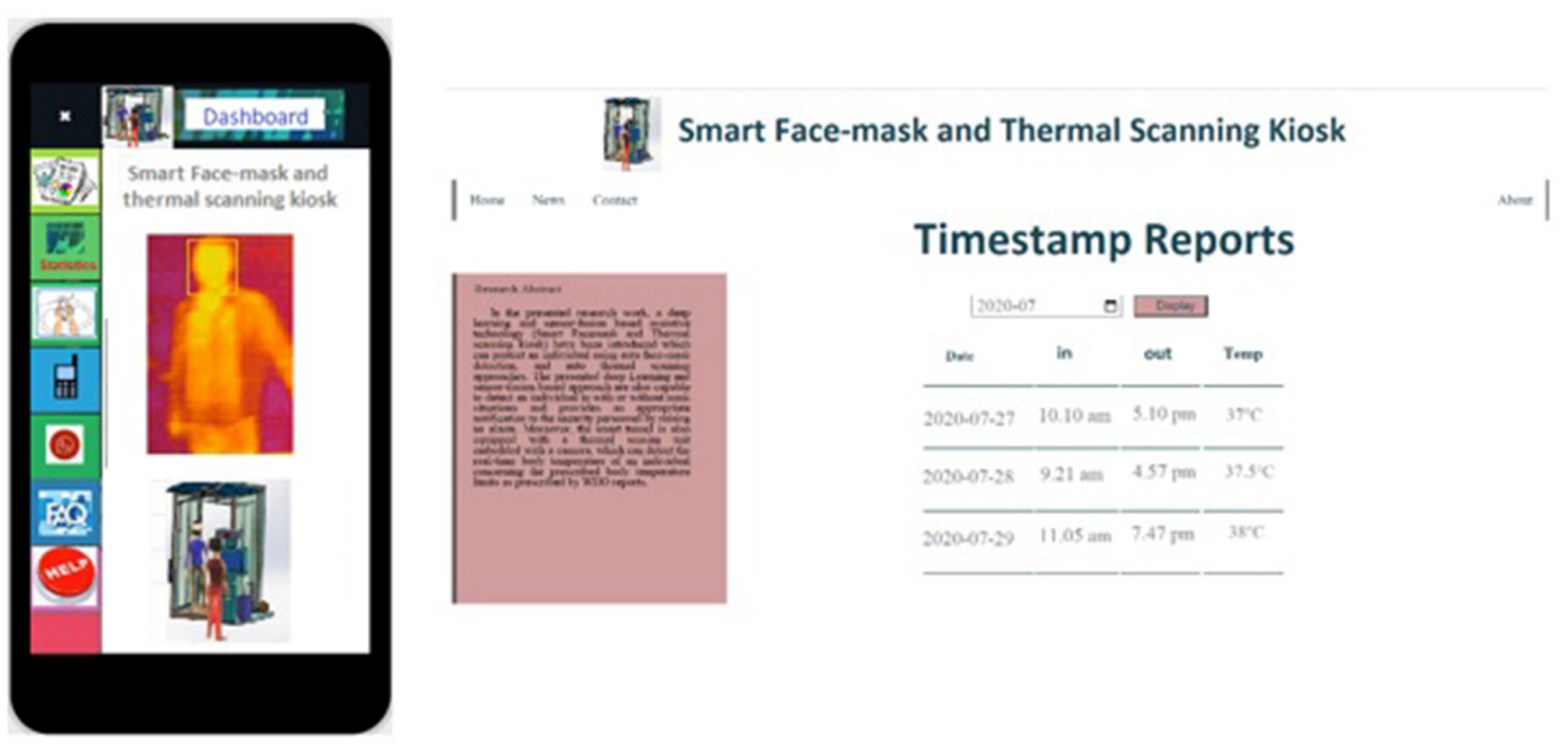
The web and mobile interface of a smart face-mask and thermal scanning kiosk.

In the conducted experiments, we have performed real-time testing of more than 800 individuals to validate the performance of the presented body temperature detection methodology. The presented methodology has utilized a Robocraze OV7670 300KP VGA camera and thermopile temperature sensor to identify individuals exceeding the prescribed standard threshold such as 98.6°F (37°C). Figure 13 represents the real-time thermal scanning results of more than 800 individuals concerning the recorded body temperature. Furthermore, it also depicts the graphical representation of the number of individuals who a VGA camera has captured from various angles. The presented thermal scanning mechanism has recorded a validation accuracy of 92.01% thermal scanning kiosk. Furthermore, the presented interfaces present the daily, weekly, and monthly reports of the counts of individuals, along with in-out timestamps fetched from the google firebase cloud computing platform. Furthermore, it also keeps track of the consumed power and also provides information on power usage reports.

### 7.3. The Web and Mobile Interface of a Smart Face-Mask and Thermal Scanning Kiosk

Figure 13 represents a GUI-based web and mobile interface design of the presented smart face-mask and thermal scanning kiosk. The presented interfaces present the daily, weekly, and monthly reports of the counts of individuals, along with in-out timestamps fetched from the google firebase cloud computing platform. Furthermore, it also keeps track of the consumed power and also provides information on power usage reports.

## 8. Conclusion and Future Enhancements

This study presents a deep learning and sensor-fusion-based assistive technology (Smart Facemask and Thermal scanning kiosk) to detect individuals with and without mask conditions. The presented research work is an extended version of the smart epidemic tunnel presented previously. In the undertaken study, the presented smart face-mask and thermal scanning kiosk have facilitated two essential tasks (i) the bifurcation of the people based on a variety of face mask conditions such as with and without face mask conditions using a YOLOv4 computer vision algorithm deployed in an NVIDIA Netson controller. In addition, (ii) it can also detect the body temperature of individuals present in the projection areas of a Robocraze OV7670 300KP VGA camera using a Thermopylae temperature sensing unit. The investigation results validate the performance evaluation of the presented smart face-mask and thermal scanning mechanism. The major findings of this study are as follows: (i) The presented system can detect an outsider who is entering the building with or without mask conditions and notifies the security control room by raising appropriate alarms. (ii) The presented smart face-mask detection and thermal scanning kiosk can function using solar energy during the day, and it functions using a solar power bank at night. This functionality has been provided by an LDR sensing unit placed in a tunnel. (iii) The presented smart kiosk also provides the facility to do real-time timestamp-based attendance of all the staff entering the smart tunnel, along with thermal scanning of the body temperature and face-mask detection. Based on the experiments, the presented YOLOV4 methodology recorded face-mask detection training and testing accuracy of 97.17 and 96.12%. Furthermore, the presented thermal scanning mechanism has recorded a validation accuracy of 92.01%. (iv) In the end, web and mobile interface has been designed to provide daily, weekly, and monthly reports of the recorded body temperatures of individuals, along with in-out timestamp values. In the future, the proposed research work can be extended with various scenarios, such as obscured faces, half, or fully obscured human faces with plastic, leather, or any other materials.

## Data Availability Statement

The raw data supporting the conclusions of this article will be made available by the authors, without undue reservation.

## Ethics Statement

Written informed consent was obtained from the individual(s), and minor(s)' legal guardian/next of kin, for the publication of any potentially identifiable images or data included in this article.

## Author Contributions

SP and AS: conceptualization and data collection and interpretation and data curation. SP, AS, and NS: manuscript editing. All authors contributed to the article and approved the submitted version.

## Conflict of Interest

The authors declare that the research was conducted in the absence of any commercial or financial relationships that could be construed as a potential conflict of interest.

## Publisher's Note

All claims expressed in this article are solely those of the authors and do not necessarily represent those of their affiliated organizations, or those of the publisher, the editors and the reviewers. Any product that may be evaluated in this article, or claim that may be made by its manufacturer, is not guaranteed or endorsed by the publisher.
